# Playing well with others: lessons from theatre for the health professions about collaboration, creativity and community

**DOI:** 10.1007/s10459-024-10314-6

**Published:** 2024-02-27

**Authors:** Julia Gray, Carrie Cartmill, Cynthia Whitehead

**Affiliations:** 1 Centre for Critical Qualitative Health Research, University of Toronto, Toronto, Canada; 2 The Wilson Centre, Temerty Faculty of Medicine, University of Toronto, and University Health Network, 200 Elizabeth Street, 1ES-559, Toronto, ON M5G 2C4 Canada; 3 Department of Family and Community Medicine, University of Toronto, Toronto, Canada; 4 Women’s College Hospital, Toronto, Canada

**Keywords:** Collaboration, Interprofessional education, Conceptual analysis, Health humanities, Performance, Theatre

## Abstract

Despite collaboration among different professions being recognized as fundamentally important to contemporary and future healthcare practice, the concept is woefully undertheorized. This has implications for how health professions educators might best introduce students to interprofessional collaboration and support their transition into interprofessional, collaborative workplaces. To address this, we engage in a conceptual analysis of published collaborative, interprofessional practices and conceptual understandings in theatre, as a highly collaborative art form and industry, to advance thinking in the health professions, specifically to inform interprofessional education. Our analysis advances a conceptualization of collaboration that takes place within a work culture of creativity and community, that includes four modes of collaboration, or the ways theatre practitioners collaborate, by: (1) paying attention to and traversing roles and hierarchies; (2) engaging in reciprocal listening and challenging of others; (3) developing trust and communication, and; (4) navigating uncertainty, risk and failure. We conclude by inviting those working in the health professions to consider what might be gleaned from our conceptualization, where the embodied and human-centred aspects of working together are attended to alongside structural and organizational aspects.

## Introduction

Despite collaboration among different professions being recognized as fundamentally important to contemporary and future healthcare practice, the concept is woefully undertheorized. The ways different professions collaborate in practice has implications for how students within distinct professional training programs can be supported and taught to collaborate interprofessionally. When and how interprofessional collaboration (IPC) might be best introduced to students (for example at the undergraduate or graduate levels), as well as best ways to support students to transition into interprofessional collaborative workplaces, are among some of the challenges faced by those working in health professions education (HPE) (Paradis & Whitehead, 2018). Paradis and Whitehead (2018) advocate for in-depth theorization of interprofessional education (IPE), including collaboration, recognizing the importance of how different groups are introduced to learning and working together, as well as the different roles and hierarchies within teams. They argue “[a]nchoring education for collaboration in more robust theories of how professions actually come together will most certainly improve the empirical success of such programs” (p. 1460). We additionally suggest it is important to consider how collaboration might be facilitated relationally among practitioners, as well as how particular workplace cultures might support or hinder the ways collaboration happens.

Analysing how the concept of collaboration is understood and enacted in different fields of practice can be an effective way to build new understandings as part of improving IPC and IPE. Theatre, as a professional practice and a field of study, is arguably one of the most collaborative art forms. Theatre requires practitioners with different training and professional parameters to work together towards a common goal: to create a theatrical production for the public. There is a history of engaging theatre and performance practices in HPE for practical skill improvement, such as role-play to support communication; there has been less interest in what conceptual knowledge and practices of collaboration in theatre might bring to the health professions.

We are an interdisciplinary Canadian writing team, including a performance studies and health humanities scholar and theatre professional (JG), a public health epidemiologist and qualitative researcher with experience in IPE within the health professions (CC), and a MD-PhD education scientist who practices and teaches in team-based primary care (CW). The interdisciplinary nature of our writing team was a strength, as our different disciplinary traditions and commitments meant that we each brought different conceptual and axiological capacities to our analysis. We welcomed interpretive tensions as part of developing nuanced conceptual understandings of collaboration.

We recognize that “collaboration is shaped by its context”, and that “knowledge, skills, behavior, attitudes and identities are all context-dependent” (Paradis et al., 2017, p. 870). This is relevant as we aim to speak across major fields (theatre and HPE), and some of our analysis cannot be translated across those fields. As such, we are not aiming for a one-size-fits-all collaborative model; rather our goal is to draw on conceptions and practices from theatre to extend thinking in the health professions, specifically how this might inform IPE.

To be clear, we are not holding theatre up as a collaborative utopia. Our aim with this article is not to romanticize theatre as *hip* or even as an *ideal* way for healthcare professionals to aspire to work. We also do not consider ways that different healthcare practices, such as surgery, might be conceptualized as a performance, as an enactment of professional skills or roles (i.e., Brodzinski, 2010; Kneebone, 2016; Mermikides, 2020). We focus our analysis on the collaborative interrelationships among theatre *practitioners*, and do not consider the collaborative aspects of the performance event itself with a live audience present. Important parallels can and should be drawn between how theatre and healthcare are both public-facing, with work involving collaboration with patients and audiences as members of that public; however, this moves beyond the scope of this article.

Our paper unfolds thus: first we discuss the discipline and practice of theatre, including the adjacent scholarly area of performance studies, as the frame for our exploration of collaboration. We then introduce our methodology, specifically a humanities-based approach called *conceptual analysis* (Haueis & Slaby, 2022; Kölbel, 2023; Walker & Avant, 1988). Next, we provide an overview of the ways theatre and drama practices have traditionally intersected with HPE. We then advance a conceptualization of collaboration that takes place within a work culture of creativity and community. We additionally articulate the ways theatre practitioners collaborate within this creative and community-oriented work culture through four interrelated *modes*, including: (1) *paying attention to and traversing roles and hierarchies*; (2) *engaging in reciprocal listening and challenging of others*; (3) *developing trust and communication*, and; (4) *navigating uncertainty, risk and failure*. Finally, we conclude by inviting those working in the health professions to consider what might be gleaned from our conceptualization of collaboration, where the embodied and human-centred aspects of working together are attended to alongside structural and organizational aspects.

## Why theatre?

Theatre is a practice-based art form and professional industry; it is also a discipline that is studied within university, college and conservatory-style settings. Theatre involves the enactment of dramatic narrative on-stage by people in space and time, and involves audiences who share in the story being told (Brook, 1993). In considering theatre, it is also useful to draw on the scholarly field of performance studies, which is an interdisciplinary academic field that brings together sociology, areas related to culture (e.g. anthropology and/or cultural studies) and the practice-based arts (such as drama, theatre and film). Performance studies is concerned with dynamic understandings of the world, and draws on the interrelatedness of space, time, bodies/embodiment, actions (what people do), broader contexts (e.g. social, cultural, and historical values and structures), sensory/emotional/imaginative aspects, and other people, to explore and make sense of a given topic (Schechner, 2004).

Theatre involves multiple roles within the overarching practice—actors, director, playwright, designers (set, costume, sound), stage management, technicians, and front of house staff, such as ushers—and each of these roles has distinct work practices that come together for the *performance event itself* (the live play). The live performance is itself collaborative in that it involves multiple people (artists, technicians, audience members) agreeing to engage in the event (Brook, 1993). However, more than the performance event itself, the *process to create* the performance is also collaborative. This includes the *rehearsal process*, a unique, collaborative process that can be both highly structured and adaptable as needed to support the preparation of the play, and *technical rehearsals* involving moving from the rehearsal hall into the theatre to organize lights, sound, costumes and other technical aspects in preparation for an audience. It is this collaborative process of creating the performance among practitioners that is the focus of this article.

## Our approach

We mobilized our aims by engaging in a *conceptual analysis* of collaboration as understood and practiced in theatre, to inform the health professions. As a humanities-based research approach, the aim of conceptual analysis is to illuminate and refine a particular concept through in-depth analysis. As such, we approached our analysis of the concept of collaboration by identifying how theatre practitioners *articulate* their understandings of collaboration and also *enact* these understandings through their practices (Haueis & Slaby, 2022; Kölbel, 2023). From nursing, Walker and Avant (1988) discuss an aligned process called *concept synthesis*, as a careful examination to acquire new insights about a phenomenon, based on empirical data including academic literature, case studies and personal/professional experience (p. 54).^1^ In this way, while we bring *conceptual analysis* to HPE from the humanities, *concept synthesis* helps us frame our approach as empirical given Walker and Avant’s articulation of engagement with specific data.

Our paradigmatic moorings in the social constructionist tradition, combined with conceptual analysis as a humanities-based methodology, shifts the emphasis of our work towards the analytic process over the prioritization of adherence to procedures for collecting and analyzing measurable empirical data. Following Eakin and Gladstone (2020), our process focuses on a “value-adding” analysis, which includes the engagement of interpretation for theorization, and the creative presence of the researchers. Through our approach we aimed “to construct out of grounded empirical data general concepts that characterize findings at a more abstract level” which we hope can be applied to other aligned phenomena (p. 2). In this way, our approach finds rigor not in reproducibility, but in the congruence and interrelationships among the study aims, theoretical depth, richness of data, and nuance of analysis in context with the enablement of readers to judge extended applicability.

To begin, we conducted a literature review. We engaged a librarian with HPE experience in searching Ovid MEDLINE and Embase for literature where theatre and/or drama practices intersected with HPE, paying attention to collaborative practices, or practices that supported collaboration. Search terms included those related to education within the health professions, such as “students, health occupations”, “med* students”, “nurs* students”, those related to the performing arts, such as “performing arts”, “dancing”, “theater”, “clown*”, and those related to interprofessional practice, such as “interdisciplin*”, “interprofess*”, “multidisciplin*”, and transprofess*”. Appendix 1 provides the detailed search strategy that was used.

Each of these articles was read in detail to determine fit with the research focus. After removing duplicates, we started with 94 articles from this combined database search. Articles were excluded if they did not contain content related to three domains: health professions education, performing arts, and interprofessional collaboration. After applying these inclusion criteria, 17 articles were included in our analysis. A reference and citation search through Web of Science was conducted with articles where there was good fit with our research focus. 24 articles were included in the Web of Science reference and citation search, leading to 4 additional articles being included in our analysis.

We additionally sought theatre-specific literature through a Google Scholar search where the word *collaboration* was in the title or a keyword. These searches were augmented by a snowball approach (Boell & Cecez-Kecmanovic, 2014) where we identified additional literature through reference lists. The abstracts and summaries for all identified literature were read; full articles and chapters that fit our criteria and contributed to our analysis of collaboration were read and re-read in-depth. First author Gray also drew on examples from her professional theatre experience.

To advance our analysis, we read and re-read all identified literature across different disciplines multiple times, noting how the word *collaboration* and aligned language was included, or not, to describe the ways people worked together in professional settings. Data were organized into broad categories, or what Walker and Avant describe as “clusters” (1988, p. 54) (e.g.: *paying attention to bodies and emotions* and *organized processes and structures*). Language used to describe collaborative theatre processes in the literature continued to be grouped into these categories, and new categories were developed and refined as needed to clarify how theatre practitioners were conceptualizing collaboration, and also how they described enacting these through their practices. Gray drew on examples from her professional practice that aligned with what was discussed in the literature, and made note where her experience diverged. In this way, we engaged in an *abductive* analytic approach, as we moved back and forth between different data, applying a theatre and performance theory lens, inviting tensions and surprise to inform our process (Cartmill et al., 2023; Tavory & Timmermans, 2014; Timmermans & Tavory, 2012).

Categories were further refined with continued attention to the relationships between the categories; careful attention was paid to the words used to describe the categories, and their interrelationships, understanding that labelling the concept must reflect its scope and nuance. This analysis was verified through a continued return to the data, including academic literature, and examples from Gray’s professional theatre experience, to ensure the concept of collaboration was empirically supported.

## Theatre and performance in health professions education

The vast majority of academic literature that cross theatre and/or drama with HPE focuses on instrumental uses of different performance practices to improve an aspect of healthcare practice (e.g. Forum Theatre, or role play). For example, there is significant literature that addresses interrelationships or improved communication between healthcare providers and patients/families (Dennis et al, 2019; Khanlou et al, 2022; Kim, 2021; Long & Gummelt, 2020; McGrath et al, 2022; Neilson & Reeves, 2019; Pastor et al., 2018; Piccoli et al., 2007; Sargean et al., 2011; Wada et al, 2019; Wesner & Chen, 2020). There is also some emerging literature that involves engaging the performing arts for healthcare students and professionals to understand client and family perspectives, or to develop empathy (Matharu et al., 2011; Reeves et al., 2021; Seko et al., 2022). While this literature mostly focuses on improving a relational aspect of healthcare practice through the application of performance, it does not directly address collaboration among healthcare professionals of different disciplines, nor draw on theories of theatre or performance as part of interpretation of study results.

There is some emerging literature about engaging drama and theatre techniques to address collaboration, interprofessional working, and teamwork in healthcare education. As one example, Lunden et al (2017) conducted a qualitative study exploring the use of Forum Theatre to foster team work and collaboration in the field of radiology. Participants from three professions, including radiology, engaged in a Forum Theatre workshop. Forum Theatre is an approach that aims to actively engage audience members (or *spect-actors*) in what happens on stage (Boal, 1985). As part of attempts to critically engage audiences to *act* in new ways within challenging situations beyond the performance, spect-actors are invited to stop the actors performing, and suggest, or even try themselves, different options. For this study, Lunden et al (2017) found these performance practices provided a dynamic learning environment for participants to engage at a “deeper level” and to experience a range of communication strategies (p. 334). While this study focuses on particular performance strategies as tools to help guide interprofessional curriculum development, they do not provide conceptual insights into how theatre practitioners think about and enact collaboration in their professional work.

From the adjacent fields of Health Humanities, there is some writing about the integration of performance approaches into health sciences education. This literature focuses on epistemological tensions in integrating humanities and arts approaches into a health sciences program (Tsampiras, 2018), as well as the ways that creative approaches can help address interdisciplinary and intercultural competencies (which Fahnert cites as being increasingly important to support students in global contexts), with a focus on problem-based learning (Fahnert, 2017). These onto-epistemological differences between performance and healthcare are well noted (e.g., Gray & Kontos, 2019; Hooker & Dalton, 2019; Mermikides, 2020), and are in part what we aim to leverage through this article.

Simulated patients is a prominent area where performance and HPE overlap. Like the aforementioned articles, most literature related to simulated patients in HPE cites improvements in person-centred or relational aspects of care as a goal. Simulated patients (also known as standardized patients) are individuals who are trained to act as a “real” patient, who simulate a particular set of symptoms or problems in order to support the training and evaluation of healthcare professionals (Williams & Song, 2016). Simulated patients have been employed in medical training (Betancourt Galeano et al., 2016; Cerdio Domingues et al., 2022; Wada et al., 2019), in the rehabilitation sciences (Dennis et al, 2019), and nursing (Edwards et al, 2018; Jennings et al., 2020), among others. This literature describes how simulated patients can support the human- or patient-focused aspects of healthcare training, including attending to patient and family emotions, improving practitioner confidence interacting with patients, as well as communication skills. Engaging simulated patients, who are often professionally trained actors, is not the same as analyzing collaborative theatre practices to better understand the concept of collaboration.

Although IPC involves different professional roles working together, we found very little literature related to how theatre and related practices might help to address these differences. There were three notable exceptions. The first are two studies that looked at the use of Forum Theatre practices for nursing students to address power dynamics in practice. Carter and McMillan-Bohler (2021) studied the ways Forum Theatre might support nursing students (many of whom were racialized) to recognize and respond to microaggressions in healthcare interactions. Given Forum Theatre’s roots in addressing social inequities, including power differences and hierarchies, this approach seems apt given microaggressions are part of maintaining oppressive social hierarchies. The authors found that the performance workshop allowed for students to identify and practice responses to microaggressions. Van Bewer et al (2021) additionally engaged Forum Theatre with nursing students to address racism, oppression and social justice with similar results.

The third study of note engaged Verbatim Theatre as a means for medical and nursing students, as well as junior health professionals, to identify and address mistreatment in hierarchical healthcare workplaces. Verbatim Theatre is a documentary approach to theatre in which the script is created using only the words of informants *verbatim* (Brown, 2010). Dalton and his team (2020) interviewed 30 healthcare students and professionals, and edited transcripts were crafted into a verbatim play called *Grace Under Pressure*. In an evaluation of the play’s impact, the authors found that participants recognized their own experiences of workplace culture in the play, which facilitated critical reflection and insights, including possible avenues to address bullying and harassment.

These exceptions aside, the ways that collaboration is a creative and communal endeavor is unexplored. Literature in this area has yet to address the importance of attending to different working roles and hierarchies as part of working with others, as well as the importance of respectfully challenging and being receptive to other people’s ideas. What also needs attention is the centrality of risks and vulnerability in theatre and healthcare practices, and the ways these can be navigated with other people. Theatre practice and performance theories can help us think through collaboration, and implications for IPE.

## Conceptions and practices of collaboration in theatre

**Fig. 1:**
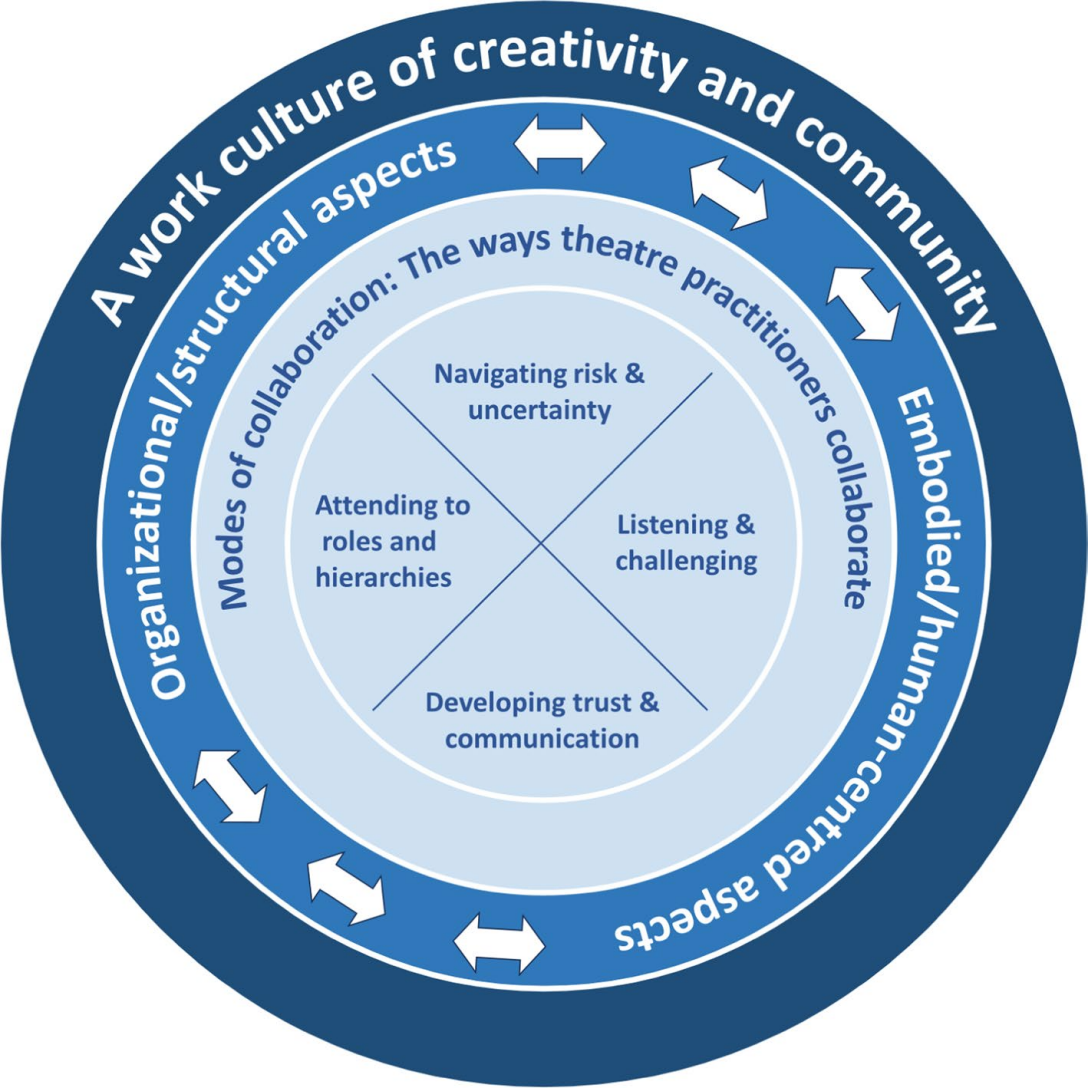
Conceptions and practices of collaboration in theatre

Through our analysis of theatre-specific literature we identified that theatre collaboration takes place within a work culture of creativity and community. This is constructed with attention to two aspects: (1) organizational/structural, and (2) embodied/human-centred. We additionally identified four interrelated *modes*, or ways that theatre practitioners engage in collaborative practice: (1) paying attention to and traversing roles and hierarchies, (2) engaging in reciprocal listening and challenging of others*,* (3) developing trust and communication, and (4) navigating uncertainty, risk, vulnerability, and failure. To support these discussions, we refer to two different theatre approaches; (1) a more traditionally-structured theatre with a specific hierarchy of roles embedded in the process (such as a director, dramaturg, actors, stage manager, etc., as discussed earlier), as well as (2) an avante garde approach called *theatrical devising* which rejects traditional structures and processes to focus on collaborative theatrical creation among participants. Figure 1 provides a schematic of these conceptions and practices of theatre.

### A work culture of creativity and community

We analysed ways that collaborative theatre practices thrive when creativity is fostered, and when community is built to support social cohesion. Collaboration can be understood as a creative process; creativity is any act that changes an existing area or process into something new (Csikszentmihalyi, 2015). American dramaturg and teacher, Lynn Thomson discusses how “[a]s a creative process, collaboration requires a high tolerance for open spaces, advanced skills for uncertainty, a hunger for the question, and a commitment to surpass what is routine” (2003, p. 120).

Creativity education scholar and researcher Daniel Harris (2014) reminds us that creativity is not a singular act, but rather it takes place in context, within particular structures and with particular people. Creativity “becomes exemplary of a series of acts, performed in embodied and disembodied ways, which eventually lead to thoughts, values and ritual practices culturally constituted as ‘creativity’” (2014, p. 4). With their professional foundation in theatre, Harris suggests a continued mooring to creativity’s performative, embodied and aesthetic roots within organized structures (p. 4). By this we understand Harris to mean that there is a push for standardized or reproducible approaches to creativity, such as mechanical, structural, or digital means (what Harris terms “disembodied”, but we term “structural/organizational”) which have come to dominate much of the creativity landscape; However, attention must also be paid to emotional, sensory, gestural and active ways that creativity happens among different people (as “embodied”). Together and when repeated over time, these disembodied and embodied means enact a particular culture of creativity. If collaboration is creative, then a *creative workplace culture* can be nurtured through attention to structural *and* embodied aspects, for people to work together towards something new.

Collaboration also thrives when practitioners feel connected within a socially cohesive community. As Thomson discusses: “collaboration is a verb not a noun, a process of engagement… The [theatre] process fosters a community of makers, who engender a shared vision, which in turn fuels… [theatrical] creation” (2003, p. 118). In the face of concerns that collaboration eradicates individual contributions or requires an absence of leadership, Thomson suggests that “collaboration does not diminish, but rather releases and expands what is unique to any [individual] artist”, and we might assert, unique to any individual professional (p. 119). Acts of working together, doing things with others who are aligned in goals or outcomes but who work within different parameters, not only hold the potential to extend beyond what can be accomplished by one person, but also build connections and link people to collective achievement and meaning. In this way, difference is recognized as important for social cohesion, and for collaboration.

### Organizational and structural aspects

Traditional, professional theatre in North America is a highly organized practice, with a series of distinct roles that execute specific tasks and processes. For example, once a theatre production is ready to be performed after a period of rehearsals, it moves from the rehearsal hall into technical rehearsals in the theatre building. Technical rehearsals are an opportunity for the artists to put together the many different aspects of the production, on and behind the stage—e.g. how the lights will change from cue to cue, how costume changes will take place, organizing where people move back stage between scenes, etc. Technical rehearsals involve very long days and are structured with the aim of being efficient, understanding there are significant financial implications for every day working in the theatre when there is no paying audience. Tensions can run high given the need to work efficiently, while balancing safety and storytelling needs. Technical rehearsals are one example of the kind of structure and organized process in theatre where collaboration takes place within a creative and community-oriented work culture.

### Embodied and human-centred aspects

The organizational aspects of the theatre production work in balance with the embodied and human-centred aspects. Theatre is a professional practice about human stories, including emotions and actions of people embedded within complex worlds (cultural, social, within a historical moment). Given these aims, organizational structures are meant to guide people to do their embodied work, and there is no attempt to assume objectivity, or to remove people themselves from the process. People and their relations are precisely the point and recognized as important.

Given this confluence of structural and embodied/human-centred aspects of theatre, power dynamics and hierarchies are thus inevitable. At its most problematic, this has historically manifested in issues that are increasingly receiving global and cross-industry recognition in the wake of the #MeToo and Black Lives Matter movements, among others (Rudakoff, 2021; Thompson, 2021, 2023). However, theatre practitioners also write openly about the need to address the interrelationships of roles, hierarchies, and even power dynamics, while working collaboratively.

## Modes of collaboration

We consider four interrelated *modes of collaboration,* or ways that theatre practitioners collaborate. We recognize that in practice these modes are not distinct; we separate them here for heuristic purposes.

### Paying attention to and traversing roles and hierarchies

Theatre practitioners are acutely aware of the importance of recognizing different roles and hierarchies while working collaboratively. This manifests in two main ways: (1) exploring hierarchies and “status” *artistically*, as the fodder for theatrical stories, and (2) understanding how different roles work collaboratively *within a working process*, to create a theatrical production.

In recognizing the importance of hierarchies in theatrical collaboration, Thomson centrally addresses this within her graduate teaching for dramaturgs. In professional theatre, a dramaturg works with other theatre practitioners to consider research and ideas underpinning a text/play (Rudakoff & Thomson, 2002). Thomson turns to the theatrical improvisation techniques of Keith Johnstone as part of teaching about interprofessional collaboration and hierarchies (Thomson, 2003, p. 117). Improvisation is an unscripted performance technique that can support actors (and others) to explore and tell stories. Key to this approach is attending to and interrogating “status” between people by making and accepting “offers” (Johnstone, 1979).

When developing his teaching approach in the 1960s, Johnstone observed that actors were not developing interesting story ideas or interactions through improvisation: “the conversations they acted out were nothing like those I overheard in life” (1979, p. 33). He began to instruct performers to be aware of their own and their improvisation partner’s status (or the status of their characters). He advised that the *differences* between improvisational partners could be *used in strategic ways* to create dramatic tension, humour, and authentic stories (p. 33). The assumption here is that differences in social roles are not something to be placated, but rather recognized, explored and engaged with when creating stories: *this* is the artistic fodder. As Johnstone notes: “Normally we are ‘forbidden’ to see status transactions except when there is a conflict. In reality status transactions continue all the time” (p. 33). It is these “status transactions… that govern human relationships” and Johnstone argues that the ability to recognize these transactions and status differences can be taught (p. 72).

As part of exploring and navigating status transactions, Johnstone suggests making and accepting “offers” which can be understood as anything an actor does to move the story forward (1979, p. 92). Offers can be accepted by a partner (“why yes, I will accept your offer that we are on an imaginary beach”) or blocked. Blocking prevents the action of the story from developing, or eradicates the partner’s idea. For a story to advance, both partners must work cooperatively to accept and build on each other’s ideas, recognizing and playing with status differences.

Thomson draws on Johnstone’s improvisational exercises to help her dramaturgy students recognize differences in *status* (e.g. working with a production’s director), and to develop skills to accept an *offer*. “The skilled collaborator… is sensitive to status and understands how to alter it, in order to remove blocks to communication” and advance the work (p. 126). This advancement does not involve taking over another’s contributions, nor consistently deferring to their offers. As Johnstone insists, collaboration is not competitive and does not exploit (1979, p. 93). It involves the development of self-awareness to understand one’s tendencies (to lead/over-take or follow/defer), and to learn to be both receptive to and challenge a working partner’s offers.

### Engaging in reciprocal listening and challenging of others

Theatre collaborators aim to contribute to the forward movement of work by balancing a receptivity to others’ ideas without succumbing to “giving up or giving in” (Thomson, 2003, p. 123). As Thomson discusses, the balance to receptivity without collapse requires a critically-informed resistance or challenge without blocking. This requires attention to another person’s ideas—or, their *offer*—as well as one’s own ideas as part of learning to develop plans, address problems creatively, and work together. Maintaining one’s own point of view at all costs, or working to sway another, is discouraged. Rather, what is important is to invite change without losing yourself, and to challenge others to consider their assumptions without demanding submission.

American theatre director Anne Bogart extends these ideas around different roles working together by discussing the theatre director. The theatre director’s craft involves interpreting a script, and building an imaginary world and story which will be played for an audience (Mitchell, 2009). Bogart advocates to think of a particular role as a “window” in that a person can view one’s own work in a particular way, and develop particular skills to advance that work (Bogart & Gay, 2014, p. 214). Bogart recommends recognizing the different “windows” and aligned skills that each professional brings, including one’s own. She argues that “*the specific window* through which we look determines *how* we look” (p. 213, emphasis added), but also the *differences* in working, and recognizing those differences is precisely what collaborative work needs to be done well. Bogart emphasizes the importance of having the humility to know when to step aside for another’s suggestions, or even temporarily looking at the play (or problem) through their specific window. The fluidity among roles does not erase the delineations of skills and perspectives, but rather invites a receptivity to difference and learning with the goal of telling a good story.

Bogart additionally warns against the inflexible control of a territorial director. “To collaborate” Bogart attests, “one needs a strong core and a supple and flexible exterior” (2014, p. 214). While the director does provide the central interpretation and makes final decisions, Bogart assures that “this aspect of control and power can be negotiated in various ways. If all of the collaborators genuinely feel the freedom to breathe and roam around [and have agency]… they will ultimately contribute more and feel more ownership in the process and the project” (p. 214).

This agency of all collaborators is advocated for by Canadian dramaturg Urjo Kareda. As a significant part of his role as Tarragon Theatre’s Artistic Director, Kareda would read all (yes, *all*) unsolicited scripts from Canadian playwrights and offer dramaturgical feedback. This labour is unprecedented, but Kareda discusses how he viewed it as part of his way of “taking seriously” the work of playwrights (who had produced these scripts in the first place); and how as a dramaturg *he* had been taken seriously by directors and other Artistic Directors (Rudakoff & Thomson, 2002, p. 24–25). To be taken seriously, he argues, means listening to that other person, taking their suggestions (or declining them respectfully and with reason), and, through these actions, empowering them to act.

We recognize this “challenge/resistance—receptivity/humility” balance, while central to collaboration, can be challenging. Here it is useful to turn to an experimental approach to theatre making called *theatrical devising* to consider the importance of developing trust and communication.

### Developing trust and communication

Theatrical devising takes place when a group of artists collaboratively creates a new theatrical work without relying exclusively on more traditional structures, roles, and processes (Heddon & Milling, 2006; Oddey, 1994). As theatre and performance scholar Michael Reinhart notes, even within these collaborative groups that push against traditional structures, these very same traditional structures can help expedite processes and group cohesion in establishing working together (Reinhart, 2022, p. 83): “The important matter is… that group activity dynamically evolves towards greater organization through the codification of practice and the development of understanding—both of which… are founded on trust and communication” (p. 85). Given the resistance to traditional structures, developing and revisiting trust and communication is foundational to these collaborative, experimental theatrical devising groups.

Trust is integral to working together and can be understood as an “agreement” (Reinhart, 2022, p. 85). Trust is related to the final production (in that “we will create something we all agree to”), and also that the process itself will be worked through, including problems that inevitably arise (p. 86). Reinhart asserts that trust is not inherent in the devising *structure*, but rather is central to the relational and collaborative *process*, in that it occurs over time and deepens as relationships are built, confirmed by experience (p. 86). “[T]rust underwrites the individuals’ investments of attention and labour, and when trust is breached, individuals must ponder if the infraction is significant enough for them to reconsider their commitment. Understood in this sense, trust is what keeps the group together” and is foundational to stability (p. 86).

Trust can be forged and strengthened. Reinhart suggests, given the *from-the-ground-up* nature of theatrical devising, groups can engage in regular discussions about the process, develop written agreements that can be revisited, and enact performance-based activities and exercises that put discussions and written agreements into action (see also Gray et al., 2020). The importance of revisiting any discussions and written agreements cannot be understated as it provides opportunities for all collaborators to confirm alignment among the discussions, written documents, and what is being practiced through the creative process.

Communication goes hand in hand with trust and is about relaying and exchanging information, as well as being fundamental to feedback (Reinhart, 2022, p. 87). As discussed earlier, communication within devising processes happens through discussion and written documentation, as well as through performance-based embodied activities; in this way it is recognized that, beyond writing, communication is verbal, physical, emotional, sensory, and imaginative. As Reinhart notes “there is very little activity that is not recognized as a type of communication (at least perceived as having informational value), as everything… is being observed, interpreted, and responded to by other participants” (p. 87). In this way, communication is both structural/organization and embodied/human-centred, and the ways these different types of information are observed and interpreted is based on a set of collective values, even if they are implicit (Reinhart, 2022, p. 88).

### Navigating uncertainty, risk, vulnerability, and failure

There are very real risks and vulnerabilities involved in creating and mounting a theatrical production. These risks and vulnerabilities can lead to *failures* which tend to fall in line with the structural/organization and embodied/human-centred aspects of collaboration. However, it is through recognizing and traversing different roles, engaging respectfully with others (by listening and challenging), and developing trust and communication, that theatre practitioners can support the mutual navigation of uncertainty, risk, vulnerability, and even failure.

Theatre artists often discuss the importance of embracing risk, vulnerability and potential failure in exploring artistic ideas and creating theatrical stories (Bailes, 2011). It is recognized that sharing a story, emotions, or ideas is emotionally risky and vulnerable with the potential to fail in the event of rejection, including residual feelings of ridiculousness or even humiliation (Gray, 2023; Gray & Kontos, 2019; Gray et al., 2020). With this embodied understanding of failure, the collaborative aspects of risk are rooted in relationality, in that the vulnerable sharing and exposing of story, emotions, or ideas takes place with others who reciprocally respond.

Theatre is also vulnerable to organizational and financial risks. Despite the perception that the cultural industries are financially destitute, in Canada in 2017, the gross domestic product (GDP) for the cultural industries broadly was over $59 billion, which was larger than the agriculture, forestry, fishing, and hunting industries combined, at $39 billion (Statistics Canada, 2019). While there was a noted decline in 2020 due to the COVID-19 pandemic, which significantly impacted live performance in particular (Canada Council for the Arts, 2022; Statistics Canada, 2022), the economic significance of the cultural sector is not irrelevant.

Despite this relative robustness in the broader sector, theatre artists specifically struggle to keep their theatre practices and organizations afloat. With limited financial resources and public funding, Canadian theatre organizations have been known to adapt their organizational structures and working practices in attempts to streamline efficiencies and focus funds towards artists’ creativity (Hill Strategies, 2015). Due to the Canadian theatre industry’s limited resources, there is also a general lack of information and research-based insights, making navigation of this precarious and rapidly changing landscape very difficult for practitioners (Hill Strategies, 2015). Producing and creating theatre in Canada, as one national example, is financially strained, and a specific production’s financial failure is not insignificant. If a production does poorly, not only are the livelihoods of artists and arts workers at stake, but a theatre company might teeter on the edge of extinction.

Thus, there are vulnerabilities related to both structural/organization and embodied/human-centred aspects of theatre. There are financial and structural vulnerabilities related to the economic precarity and stresses of the theatre sector, and there is an emotional vulnerability in sharing stories that may not resonate with audiences. These vulnerabilities are intertwined, as if stories do not resonate with audiences, box office revenues are affected; and if an artistic project does not have sufficient financial support for development it will potentially be diminished with greater possibility of leading to poor audience response.

These risks of failure are a significant part of why building community is so important in theatre. In building community and supporting social cohesion, practitioners bolster each other through the uncertainty and failures of creating and producing theatre. As American dramaturg Norman Frisch advocates, it is through collaboration that there is not only the potential to solve problems and advance the work further, but also to develop a sense of connectedness to others. “One of the markers of healthy collaboration,” Frisch discusses, “is the sense you have of being lifted up” (Rudakoff and Thomson, p. 283). Collaboration is important because “whatever you arrive at together will be better than what you would have come to on your own” (p. 283).

## Conclusion

Our analysis identifies the ways that collaboration is relational. By this we mean that collaboration happens in particular contexts, and with particular people who are embodied, thinking, emotional, sensory beings. In the theatre profession, collaboration happens well in work cultures that are constructed to be creative and community-oriented. This work culture is built with attention to the structural and organizational aspects (such as how processes are organized), as well as embodied and human-centred aspects (such as recognizing the ways emotions, stories, and meaning are implicated in work). When work cultures are creative and community-oriented, theatre practitioners collaborate knowing the risks and vulnerabilities by paying attention to and traversing roles and hierarchies, building trust and communication, as well as reciprocally listening to and challenging each other.

There are limits to our research and analysis of collaboration. Most significantly, our research is highly context-specific—as an exploration of theatre practice as collaborative, with considerations for HPE, with most of our examples being Canadian and North American—and as such, we make no claims to generalizability. We additionally do not engage with other theories or frameworks of collaboration from HPE or IPE, nor consider nuances of how our conceptualization comes into conversation with those other frameworks, which would be an important next step. Our conceptual analysis was designed to bring collaborative concepts from theatre to the attention of health professions educators. The concept of collaboration gleaned from a Canadian context may or may not resonate with others in different contexts, and, similarly, analyses from those other contexts would be important for further idea development. Taking our analysis forward would involve piloting and embedding these ideas into curricular interventions and evaluating this implementation. Aligned with this, we do not offer concrete ways that HPE educators can use these ideas, or specific tools for engagement; rather we offer new conceptual understandings for educators. As another limitation, we cannot be certain that we found all literature related to theatre and HPE, or theatre and collaboration, as publications may have been indexed in ways that were beyond our search. For example, arts and humanities publications have traditionally been in the form of books over articles, and searching by articles misses the vast majority of books.

Despite these limits, we can begin to extend some of our analysis to healthcare practice, HPE and IPE. In healthcare, practice is inherently relational, however, emphasis is more often than not placed on individual practitioners performing tasks efficiently and in standardized ways that can be reproduced. Without diminishing the importance of standardization, including the ways “standards help translate evidence into practice, reduce harmful variation, and support equitable care for vulnerable populations” (Paradis, et al., 2021, p. 323), considering the limits here might open up thinking about what could be done differently, particularly related to collaboration. As Paradis and colleagues argue, standardized procedures are most useful when handling statistically frequent events or disease presentations, but insufficient when aiming to address more rare events, or more complex disease presentations in intersectional social worlds (p. 323). Indeed, as the authors argue, standardization “constrains how individuals interpret and tackle the diversity and uniqueness of the situations in which they are placed and of every person they interact with” (p. 323). Standardization is an important starting point, and should guide practitioners “without stifling connection, customization, and creativity” (p. 324).

The tackling of diversity and uniqueness of situations is precisely why strong collaboration is needed. This means considering how workplaces might support ways for people to engage creatively and in community, and how educators might prepare students for this possibility in future workplaces as well. In considering what healthcare might glean from theatre, we suggest that healthcare might consider the embodied and human-centred aspects of workplaces and education in closer juxtaposition to the organizational and structural. Without suggesting standardization is unimportant, can healthcare consider how traditional approaches to standardization might hinder human-centred aspects of care? Can healthcare contemplate the ways that “people and their relations are precisely the point” as part of the management of patients’ conditions and illnesses?

We recognize that many in healthcare see the importance of teambuilding, and work has been done in this area. Teambuilding activities, both inter-professionally or intra-professionally, are common practice within healthcare, and there is additional research to support this practice (e.g., Hope et al., 2005; Rosen et al., 2018). Emphasis here is often at the level of building trust and communication, which parallels our identification of this mode of collaboration in theatre. That said, we wonder if this work considers attending to power and hierarchies, reciprocal listening and challenging, and openly navigating risk, vulnerabilities and failure within community. We further wonder if these teambuilding activities attend to both structural/organization and embodied/human-centred aspects of working together. Further research is needed to consider how our conceptualization of collaboration based on theatre aligns or diverges from other work happening in healthcare practice, HPE, and IPC.

We pose several questions about how the modes of collaboration might specifically be considered within HPE and IPE. First, how might IPE address roles and hierarchies within health professions training? Following Johnstone’s work teaching improvisation, how might roles and hierarchies, or *status*, not be placated, but recognized, explored, and engaged with as part of thinking about how different professions work together? This opens up thinking around the second mode: reciprocal listening and challenging. It is in part by transparently attending to roles and hierarchies that different practitioners might reflexively listen to and respectfully challenge practitioners from other professions. How might we teach different practitioners to *accept an offer* within healthcare spaces, as part of addressing the complexities of patient care?

While it is generally recognized in HPE and IPE that trust and communication are fundamental to strong collaboration and team work, can cues be taken from theatre to consider other, non-verbal or non-text-based communication? This might include “verbal, physical, emotional, sensory, and [even] imaginative” ways of sharing information and coming to agreements about how work will take place. Perhaps educators can consider Reinhart’s assertion that trust is not inherent to working structures, but occurs over time, deepens as relationships are built, and is confirmed by experience (2022, p. 86). In this light, can IPE consider how collaboration is built through trust as an ongoing aspect of work, rather than framed as a competency to be learned, and which—once learned—can then be applied effectively across different settings and contexts.

Finally, while theatre and healthcare serve different purposes, and are rooted in different onto-epistemological traditions, practitioners in both fields navigate risk, vulnerabilities and failures. In healthcare, risk management is mostly positioned as being solved by standardized approaches. We would invite those in healthcare to consider how collaborative care and education models might keep a dual focus on the structural/organizational aspects of mitigating risks together with the embodied/human-centred aspects simultaneously.

In conducting this research, we aimed to explore how a theoretical and conceptual analysis about collaborative practices in theatre might contribute to healthcare learnings. More fully engaging with rich theoretical offerings from collaborative spaces beyond healthcare, including theatre and other arts and humanities approaches, will help us proceed beyond simply considering their instrumental uses in our settings. As healthcare professionals and educators aim to address increasingly complex practice needs, and aligned educational approaches, it will be beneficial to deeply contemplate the affordances that diverse onto-epistemologies from other disciplines, such as theatre, contribute to healthcare and healthcare education. Ultimately, working together creatively and in community, including reflecting on the importance of diverse theoretical and practical approaches, will help advance our collective aim to improve the lives of those we serve.

## Appendix 1: medline and embase literature search strategy

1. education, predental/or education, premedical/or exp education, professional/or exp inservice training/or exp schools, health occupations/(367990)
2. exp Students, Health Occupations/(82386)
3. ((med* or nurs* or therap* or "health professions" or "health profession" or pharm* or dent* or physician* or surgeon* or doctor* or clinic* or hospital* or healthcare) adj2 (student* or learn* or teach* or educat* or instruct* or train???)).tw,kf. (339009)
4. Translational Medical Research/and (student* or learn* or teach* or educat* or instruct* or train???).tw,kf. (1736)
5. exp Health Occupations/ed or exp Health Personnel/ed (220072)
6. exp "diagnostic techniques and procedures"/ed or exp therapeutics/ed or exp surgical procedures, operative/ed (28090)
7. exp Teaching/and exp health occupations/(32449)
8. exp Teaching/and exp health personnel/(16154)
9. (clinical competence/or professional competence/) and (student* or learn* or teach* or educat* or instruct* or train???).tw,kf. (71480)
10. (teach* or educat* or learn*).jw. (164449)
11. exp Patient Education as Topic/(88396)
12. patient simulation/(5434)
13. exp curriculum/and exp health occupations/(43511)
14. exp curriculum/and exp health personnel/(17647)
15. pedagog*.tw,kf. (10976)
16. or/1–15 (844402)
17. Dancing/(3339)
18. (performing adj4 (art or arts)).mp. (527)
19. danc*.mp. (9569)
20. ((performing or play*) adj4 music*).mp. (1898)
21. (((theatr* or theater*) adj6 (drama* or art or arts or play* or musical or opera* or perform*)) not "operat* theat*").mp. (1483)
22. (music* adj4 instrument*).mp. (1503)
23. (clown* or circus*).mp. (1284)
24. (music* adj4 (band* or group*)).mp. (1965)
25. (orchestra or orchestras).mp. (720)
26. or/17–25 (17589)
27. 16 and 26 (934)
28. (interdisciplin* or interprofess* or transprofess* or cross-profess* or cross-disciplin* or inter-disciplin* or inter-profess* or trans-profess* or transdisciplin* or trans-disciplin* or multidisciplin* or multi-disciplin* or multiprofess* or multi-profess*).mp. (234436)
29. 26 and 28 (289)
30. 16 and 29 (58)
31. (Teaching and Rehearsing Collaboration).m_titl. (0)

## Footnotes

1 As is not uncommon in interdisciplinary work, we recognize that similar research approaches can be discussed with different language across disciplines. This is one of those instances; as such our aim is to recognize and draw on *conceptual analysis* as based in the humanities, and *concept synthesis* as being rooted in nursing.
